# Proposed equations and reference values for calculating bone health in children and adolescent based on age and sex

**DOI:** 10.1371/journal.pone.0181918

**Published:** 2017-07-31

**Authors:** Rossana Gómez-Campos, Cynthia Lee Andruske, Miguel de Arruda, Camilo Urra Albornoz, Marco Cossio-Bolaños

**Affiliations:** 1 Faculty of Physical Education, State University of Campinas, Campinas, Brazil; 2 Universidad Autonoma de Chile, Talca, Chile; 3 Escuela de Pedagogías en Inglés, Facultad de Ciencias de la Educación, Universidad de Talca (Campus Linares), Talca, Chile; 4 Research Network on Human Biological Development, Arequipa, Peru; 5 Escuela de Kinesiología, Facultad de Salud, Universidad Santo Tomás, Talca, Chile; 6 Department of Physical Activity Sciences, Catholic University of Maule, Talca, Chile; 7 Universidad Nacional de San Agustín, Arequipa, Perú; Leeds Beckett University, UNITED KINGDOM

## Abstract

**Background:**

The Dual Energy X-Ray Absorptiometry (DXA) is the gold standard for measuring BMD and bone mineral content (BMC). In general, DXA is ideal for pediatric use. However, the development of specific standards for particular geographic regions limits its use and application for certain socio-cultural contexts. Additionally, the anthropometry may be a low cost and easy to use alternative method in epidemiological contexts. The goal of our study was to develop regression equations for predicting bone health of children and adolescents based on anthropometric indicators to propose reference values based on age and sex.

**Methods:**

3020 students (1567 males and 1453 females) ranging in ages 4.0 to 18.9 were studied from the Maule Region (Chile). Anthropometric variables evaluated included: weight, standing height, sitting height, forearm length, and femur diameter. A total body scan (without the head) was conducted by means of the Dual Energy X-Ray Absorptiometry. Bone mineral density (BMD) and the bone mineral content (BMC) were also determined. Calcium consumption was controlled for by recording the intake of the three last days prior to the evaluation. Body Mass Index (BMI) was calculated, and somatic maturation was determined by using the years of peak growth rate (APHV).

**Results:**

Four regression models were generated to calculate bone health: for males BMD = (R^2^ = 0.79) and BMC = (R^2^ = 0.84) and for the females BMD = (R^2^ = 0.76) and BMC = (R^2^ = 0.83). Percentiles were developed by using the LMS method (p3, p5, p15, p25, p50, p75, p85, p95 and p97).

**Conclusions:**

Regression equations and reference curves were developed to assess the bone health of Chilean children and adolescents. These instruments help identify children with potential underlying problems in bone mineralization during the growth stage and biological maturation.

## Introduction

The base for bone health is created during infancy and adolescence [1]. Generally, it is accepted that the proper development of bone mineral content during growth and biological maturation is key for skeletal health [2] during adult life.

Therefore, developing early skeletal deterioration could be caused by genetic factors or related disorders such as lifestyle, for example, obesity, or some medical treatments [3], and inadequate consumption of calcium and vitamin D [4]. These play a fundamental role in the deterioration of bone health in children and adolescent students. Therefore, poor lifestyle habits, such as sedentary, may hinder peak genetically programmed bone mass.

The evaluation of bone health in pediatric patients allows identification of children with low accumulation levels of bone minerals or of developing osteoporosis due to low bone mineral density (BMD) [5].

In this context, bone densitometry is widely used to evaluate bone mineral density. Its objective is to identify individuals with a risk of bone fragility in order to establish, guide, and monitor their treatment afterwards [6]. Therefore, Dual Energy X-Ray Absorptiometry (DXA) has become the gold standard for measuring BMD and bone mineral content (BMC) of children and adolescents in the entire world. This is due to its speed, high precision, safety, low radiation emission, and wide accessibility [7].

In general, DXA is ideal for pediatric use [5]. However, the increased cost for evaluations use of different software programs, and the development of specific standards for particular geographic regions [8,9] limit its use and application for certain socio-cultural contexts. Additionally, it may provide contradictory results when used by countries that do not have available national standards.

In this sense, anthropometry may be a low cost and easy to use alternative method in epidemiological contexts. Therefore, the purpose of this study was to develop regression equations to predict the bone health of children and adolescents based on anthropometric indicators. The DXA method was used as a reference to propose reference values according to age and sex. This data could provide accurate information to researchers and doctors to evaluate the state of the bones in Chilean children and adolescent students. To date, no pediatric reference database exists for clinically and anthropologically evaluating bone health during physical growth and biological maturation. Moreover, this could be crucial in preventing and controlling for the risk of osteoporosis and early age fractures prevalent with advanced age [10].

Therefore, the authors of this study hypothesize that the years of peak height velocity (PHV) based on anthropometric variables, forearm length, and femur diameter could predict the bone health of children and adolescents. In addition, it is possible that the creation of percentiles based on the LMS method could contribute to diagnosing, classifying, and monitoring BMD and BMC based on age and sex.

## Methodology

### Subjects

A descriptive cross-sectional study was conducted. The students recruited for this research were selected from 12 public schools in the Maule Region (Chile). The initial sample was comprised of 3365 students (1761 males and 1604 females) with ages ranging from 4.0 to 18.9 years. Once the study began, the sample was composed of 3020 students (1567 males and 1453 females). The number of subjects was reduced in order to exclude those students (78 males and 66 females) who smoked and those (12 males and 6 females) with one or more bone fractures three months’ prior to the research. Also, students (82 males and 66 females) taking vitamin supplements and those below the p<3 or above the >p97 based on BMI were excluded from the study [11].

This study was approved by the Scientific Ethics Committee of the Universidad Autónoma de Chile (protocol no. 238/2013). The experimental protocol was based on the Helsinki Declaration Accord (World Medical Association for Human Subjects). Informed consent forms were also approved by the university Ethics Committee.

Selected schools were visited in order to explain to parents and/or guardians, caretakers, and teachers the study’s objectives and procedures. This procedure was carried out three times resulting in a greater number of subjects in order to conduct the research. Parents, guardians, or caretakers agreeing that their children could participate in the study signed a written informed consent form on behalf of these minors. These documents were subsequently kept in a locked cabinet in a locked room at the university.

Permission to carry out the study was requested from the Dirección de Administración de Educación Municipal (Municipal Administration of Education) of Talca (DAEM-Chile) and each school’s administration in order to collect data from the respective schools. After permission was obtained from the administrative bodies, students were transported by bus from the schools (to and from) to the University Autónoma of Chile (laboratory) to be evaluated.

### Methods

The anthropometric variables and the Dual-Energy X-Ray Absorptiometry scan were carried out in a closed laboratory with a constant temperature between 20 to 24°C. All measurements were taken during the morning and the afternoon (8:30 a.m. to 12 noon and 14:00 to 18:00 hours) from Monday to Friday during the months of March to November, 2015. The evaluation of the anthropometric variables and the DXA scan lasted approximately 10 to 12 minutes for each student.

Each student’s age to the decimal was recorded (birth date and evaluation date). Additionally, standing height was measured using a portable stadiometer (Seca Gmbh & Co. KG, Hamburg, Germany) with 0.1mm accuracy based on the Frankfurt Plan. Sitting height (cephalic-trunk height) was taken while the subject was sitting on a wooden bench with a height of 50cm with a measurement scale of 0 to 50cm and a precision of 1mm. The length of the forearm (m) or the distance between the radial and styloid points were measured using an anthropometer brand Cescorf (Made in Brazil) with a scale of 0 to 60cm a 1mm accuracy. The subject assumed a relaxed position with the arms hanging by the sides. The right forearm was slightly rotated externally to a mid-pronated position. The diameter of the biepicondilar femur (cm) was measured with an anthropometer brand Cescorf (Made in Brazil) with a scale of 0 to 20cm and a precision of 1mm. The subject assumed a relaxed sitting position with the palms of the hands resting on the muscles. The distance between the two most salient points of the femoral condyles was measured. The “international working group of kineanthropometry” standardized protocol described by Ross and Marfell-Jones [12] was used to measure the anthropometric variables. All anthropometric variables were measure twice by three evaluators. The Technical Error of Measurement (TEM) for weight, standing height, and sitting height varied between 1 to 2% while the length of the forearm and the diameter of the femur ranged from 1.5 to 2.5%.

The body mass index (BMI) was calculated using the standard formula: body mass (kg)/height^2^ (m). Biological maturation was controlled for by means of the somatic maturation. It was predicted by using a regression equation proposed by Mirwald et al. [13]. This method indicates the time before or after peak height velocity (PHV). Maturity offset = –9.376 + 0.0001882×leg length and sitting height interaction + 0.0022×age and leg length interaction + 0.005841×age and sitting height interaction– 0.002658×age and weight interaction + 0.07693×weight by height ratio, where r = 0.94, r^2^ = 0.89 and SEE = 0.57. Length measurements are in centimeters, and weight measurements are in kilograms. The weight by height ratio is multiplied by 100.

For the total body scan (without the head), the Dual-Energy X-Ray Absorptiometry (Lunar Prodigy; General Electric, Fairfield, CT) was used. The scans were conducted in one laboratory with one densitometer. The bone density and bone mineral content values of the total body were taken from % of body fat, lean fat mass, fat mass, and bone mass. For this process, the subjects had to lie on a scanning platform in a supine position with the arms and legs extended (pronated). The ankles were tied together with a Velcro belt to ensure a standard position. The participants were warned about wearing jewelry and the presence of any type of metal on or in the body that could impede the scan. Ten percent of the sample studied was scanned twice (230 subjects) in order to guarantee the technical measurement error (TME). The evaluation of the total body showed a TME of less than 2.5%. The measurements were carried out by two fully experienced technicians. They calibrated the equipment daily according to the manufacturer’s instructions.

To calculate the amount of calcium in the diet, the subject’s recall of the last 3 days consumption categorized by meals at breakfast, lunch, and dinner were used. Subjects were asked about the frequency of consumption and portion size of foods and drinks. The parents of children age 11 and under filled out the report form while the adolescents 12 and older responded for themselves. Quantification of calcium consumption was analyzed by using the computer program NutWin 6.0 [14].

### Statistical analysis

The descriptive statistical analysis of the arithmetic mean and the standard deviation were calculated. The “t-test” for independent student samples was run to determine the differences between both sexes and between the DXA and predictor values. A paired t-test was conducted to compare the DXA values and the equations generated. The relationship between variables was verified by using the Pearson correction coefficient. Four regression models were developed to predict bone health (2 for BMD and 2 for BMC). The entire sample (1567 males and 1453 females) was used to generate the equations. Multiple regression analysis was carried out in steps. The objective was to identify the best combination of predictive variables of bone health. The equations were analyzed by using R^2^, SEE, and multi-co-linearity through the variance inflation factor (VIF). Lin’s approach [15] for the concordance correlation coefficient (CCC) was calculated using MedCalc Statistical Software v.11.1.0, 2009 (Mariakerke, Belgium), to verify the accuracy (A) and precision (P) between the estimated BMD and BMC values by DXA and determined by predictive model. The Bland-Altman method [16] was used to examine the agreement and trend of the differences and the averages between the reference and predictive model values for the BMD and BMC.

The statistical LMS method [17] was used to construct reference curves starting from the predictor values both for the BMD as well as for the BMC based on age and sex. The LMS technique estimates three parameters: median (M), sd (S), and power in the Box-Cox transformation (L). These three parameters vary as a function of age. Data normality was verified using the Kolmogórov-Smirnov test corrected by Lilliefors. The residue variance homogeneity was verified using the Levene test. In all the cases, p<0,001 was adopted. All analyses were carried out using SPSS software, version 10.0 (SPSS, Chicago, IL).

## Results

The variables that characterized the sample studied are presented in Table 1 below. Males showed greater weight, standing height, sitting height, length of forearm, BMD, BMC, calcium consumption, lean mass, and bone mass compared to the females (p<0.001). On the other hand, the females showed greater fat mass and fat % when compared to the males. No differences were found in chronological age and the dimension of the femur (p>0.001). Furthermore, the years of peak growth velocity (APHV) in males occurred at 14.98±0.9 years and for the females at 11.78±0.48 years. Significant differences emerged in nutrition between the males and females (p>0.001).

**Table 1:** Anthropometric, bone health, and body composition characteristics of the sample studied.

Variables	Males (n = 1567)		Females (n = 1453)		Total (n = 3020)	
	X	DE	X	DE	X	DE
***Anthropometry***						
Chronological age (years)	13.36	3.82	12.32	3.79	12.95	3.84
Biological age (APHV)	14.98	0.93*	11.78	0.48		
Weight (kg)	54.50	19.83*	47.77	16.69	51.84	18.94
Standing height (cm)	155.15	20.64*	146.47	15.52	151.72	19.25
Sitting height (cm)	81.08	10.50*	77.00	8.55	79.47	9.98
Forearm length. (cm)	24.13	3.60*	22.22	2.96	23.38	3.49
Femur diameter (cm)	9.14	1.10	8.40	0.98	8.85	1.11
***Calcium consumption (mg/d)***	732.8	398.1*	636.2	482.6	684.5	416.4
***BMD (g/cm***^***2***^***)***						
Total body	0.93	0.22*	0.82	0.16	0.89	0.21
***BMC (g)***						
Total body	1.66	0.71*	1.26	0.45	1.50	0.65
***Body composition (kg) DXA***						
Fat Mass	13.75	7.67*	16.75	8.23	14.94	8.03
Lean Mass	38.88	14.44*	29.31	9.03	35.07	13.41
Bone Mass	2.08	0.79*	1.64	0.52	1.91	0.72
% Fat	26.11	8.70*	35.19	6.41	29.72	9.04
***Nutritional status (BMI)***^***a***^	N	%	n	%	N	%
Under weight	25	1.6	31	2.1	56	1.9
Normal	898	57.30	868	59.70	1766	58.50
Over weight	342	21.80	298	20.50	640	21.20
Obese	302	19.30	256	17.60	558	18.50
Total	1567	100	1453	100	3020	100

Legend: X = Mean, SD = Standard deviation, APHV = years of peak growth velocity, BMD = Bone mineral density, BMC = Bone mineral content, BMI = Body Mass Index

* = Significant difference (p<0.001)

a = (X^2^ = 3.672; gl = 3

p = 0.2992).

**Fig 1:**
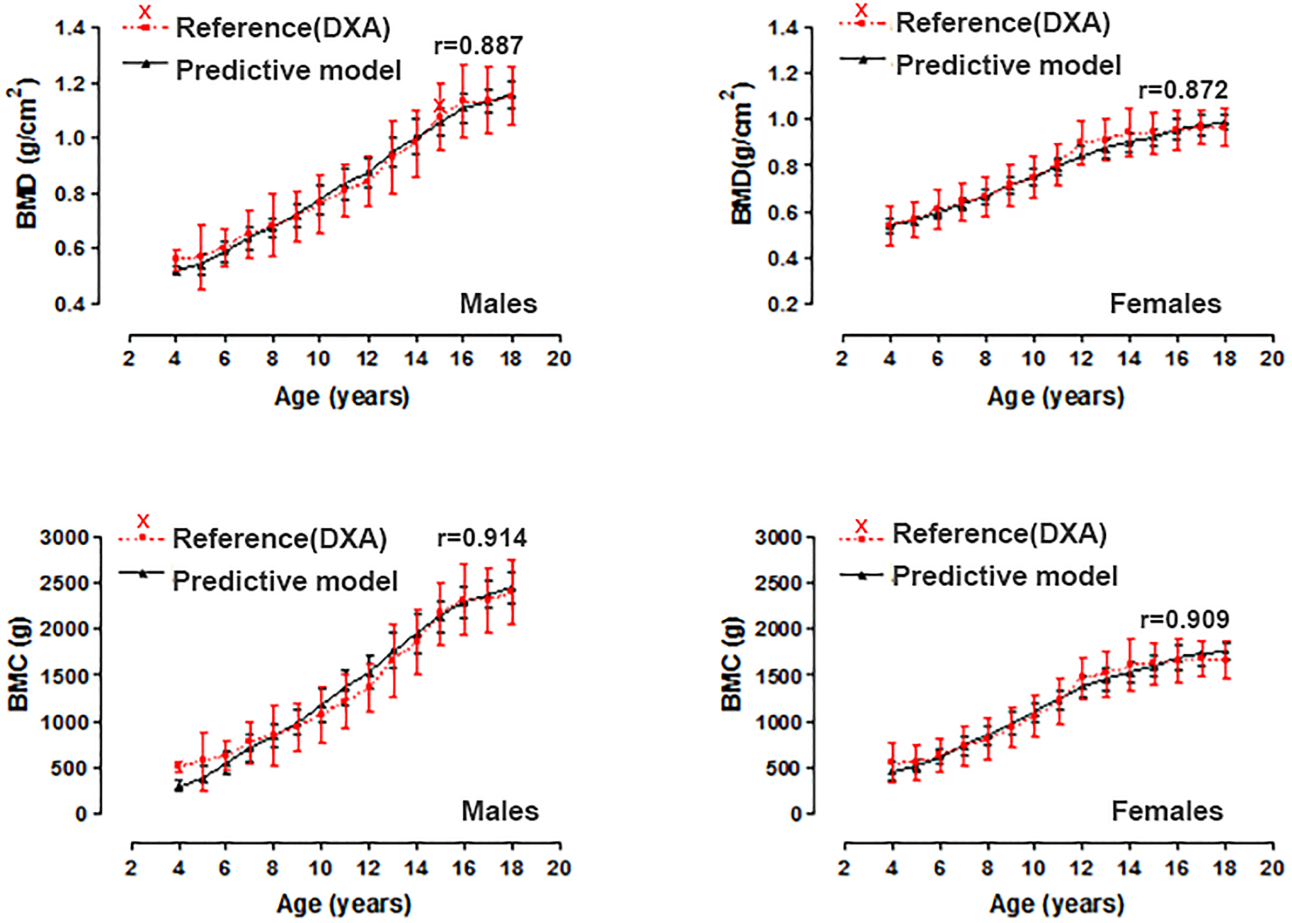
Total body value for BMD and BMC determined by DXA versus proposed predictive models based on age and sex.

The regression equations that were developed to estimate bone health (BMD and BMC) are illustrated in Table 2. The four equations showed an explanatory power of 76 to 84%. The SEE varied 0.08 to 0.29. The variance inflation factor (VIF) for the predictor variables showed values between 1.781 and 4.099. In general, the four models developed are highly significant (p<0.001). Fig 1 illustrates the BMD and BMC values found in the DXA and the values proposed by the predictor model proposal. In all cases, no significant values occurred (p>0.001). The Pearson correlation coefficient reflected values from 0.87 to 0.91, respectively.

**Table 2:** Regression equations used to estimate bone health based on biological maturation and anthropometric indicators.

n°	Equations	VIF	R	R^2^	SEE	p
	**Males**					
1	BMD = 0.605+0.056* APHV + 0.008*Forearm length + 0.022*Femur diameter					
	APHV	4.034	0.89	0.79	0.10	0.000
	Forearm length	4.099				
	Femur diameter	1.867				
2	BMC = 0.43+0.18* APHV + 0.039*Forearm length + 0.06*Femur diameter					
	APHV	4.034	0.91	0.84	0.29	0.000
	Forearm length	4.099				
	Femur diameter	1.867				
	**Females**					
3	BMD = 0.469+0.027* APHV + 0.007*Forearm length + 0.019*Femur diameter					
	APHV	3.150	0.87	0.76	0.08	0.000
	Forearm length	2.963				
	Femur diameter	1.781				
4	BMC = 0.077+0.07* APHV + 0.032*Forearm length + 0.48*Femur diameter					
	APHV	3.150	0.91	0.83	0.19	0.000
	Forearm length	2.963				
	Femur diameter	1.781				

Legend: APHV = years of peak height velocity, BMD = Bone Mineral Density, BMC = Bone Mineral Content, VIF = variance inflation factor, SEE = Standard Error of Estimate

**Fig 2:**
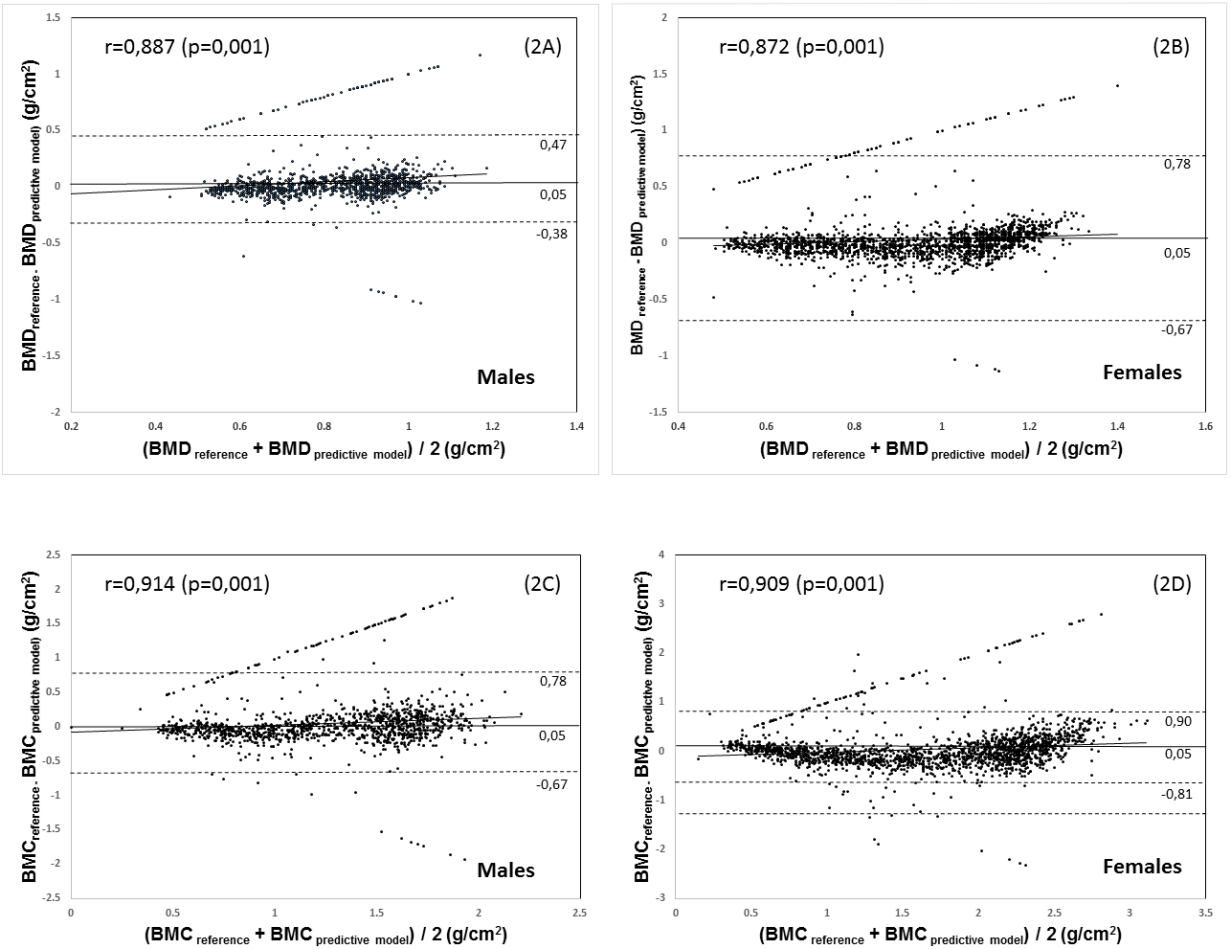
Analysis of the agreement of Bland-Altman plotting of values between the DXA reference method and predictive models for BMD (2A, 2B) and BMC (2C, 2D). The solid line: Mean Difference; dashed line: limits of agreement of 95%.

The relationship between the DXA reference and the regression equations (BMD and BMC) can be observed in Fig 2. The four equations developed showed a wide range of limits of agreement related to the reference method. For example, in males, these values varied from -0.38 to 0.47 for BMD and from -0.67 to 0.78 for BMC. For females, BMD values ranged from -0.67 to0.78, and for BMC, the values varied from -0.81 to 0.90. In general, the four equations show significantly high correlations (p<0.001).

**Fig 3:**
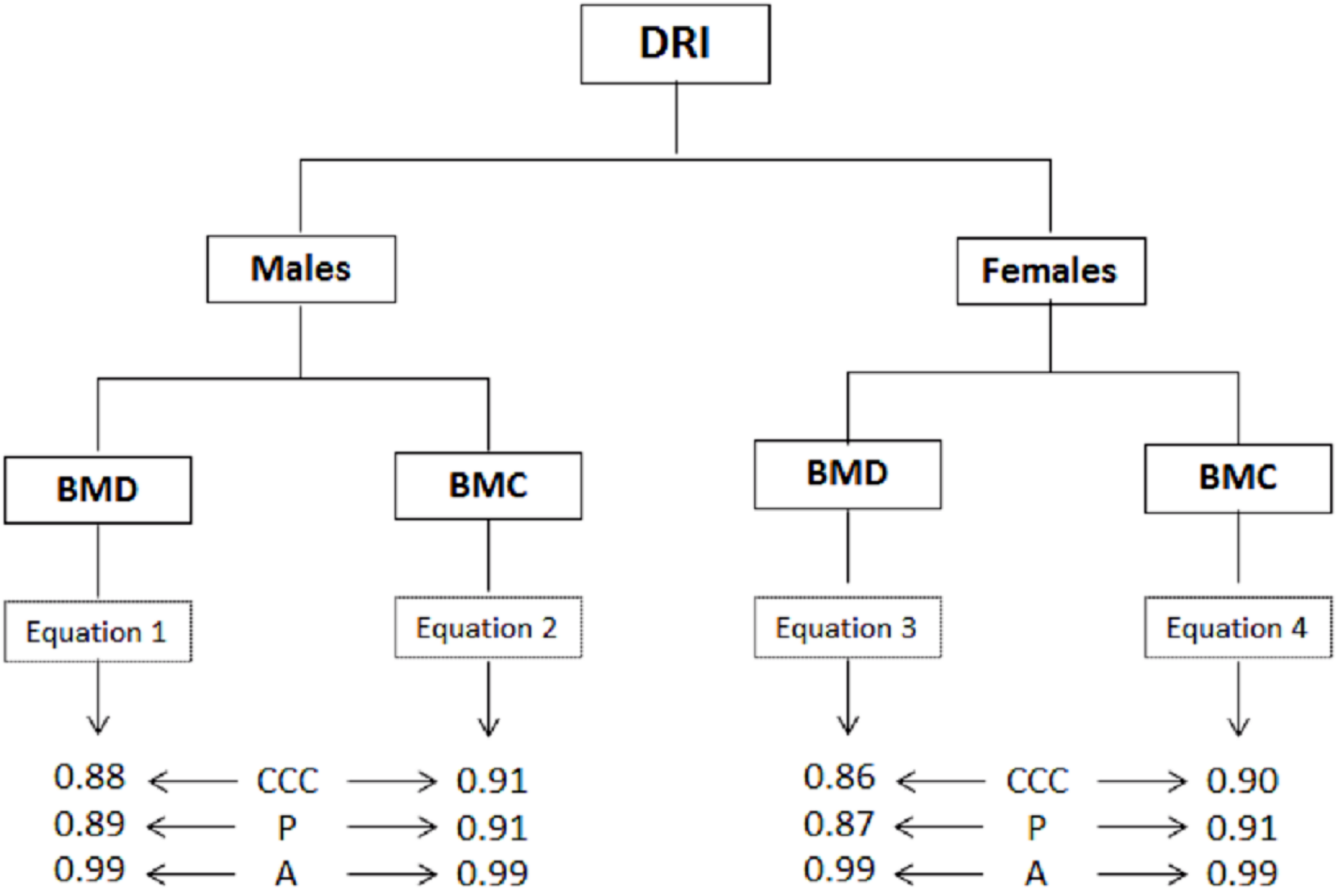
Indicator values for desirable reproducibility (IRD) that characterize the agreement between the DXA reference method and the proposed equations. Concordance correlation coefficient (CCC), Precision (P), Accuracy (A).

The IRD values used to evaluate the agreement based on the concordance correlation coefficient (CCC) in terms of precision (P) and accuracy (A) are illustrated in Fig 3. The values for the CCC for the BMD equations varied from 0.86 to 0.88 and for the BMC from 0.90 to 0.91. The precision (P) for the BMD equations varied between 0.87 and 0.89 while for the BMC, the value was 0.91. Accuracy (A) for all four equations was 0.99 (BMD and BMC).

The mean and ±SD values obtained for the DXA and regression equations are depicted in Table 3. No significant differences occurred in the comparisons or in both sexes (p>0.001). The four equations that were developed showed values similar to the reference (DXA).

**Table 3:** Mean and ±SD values for BMD and BMC determined by DXA and regression equations for both sexes.

Sex	Variables	X	SD	t	p
Males	**BMD**				
	DXA	0.93	0.22	0,0000	0.999
	Equation 1	0.93	0.20		
	**BMC**				
	DXA	1.66	0.71	0.8716	0.3835
	Equation 2	1.68	0.65		
Females	**BMD**				
	DXA	0.82	0.16	1.594	0.1110
	Equation 3	0.81	0.14		
	**BMC**				
	DXA	1.26	0.45	0.5568	0.5777
	Equation 4	1.27	0.41		

Legend: X = BMD = Bone Mineral Density, BMC = Bone Mineral Composition, *P >* 0.001 (paired *t* test).

Table 4 and Table 5 illustrate the BMD and BMC for the total body of children and adolescents from ages 4.0 to 18.9 years of both sexes. The distribution of the proposed percentiles P3, P5, P15, P25, P50, P75, P85, P95, and P97 allows us to verify the accumulation of bone health with age. These values were determined based on the equations proposed in Table 2.

**Table 4:** LMS values and percentile distribution of BMD (g/cm^2^) of the total body in children and adolescents based on age and sex.

Age	L	M	S	P3	P5	P15	P25	P50	P75	P85	P95	P97
**Males**												
4.0–4.9	-0.0074	0.4999	0.00065	0.44	0.45	0.47	0.48	0.50	0.52	0.54	0.56	0.57
5.0–5.9	-0.0089	0.5430	0.00065	0.48	0.49	0.51	0.52	0.54	0.57	0.58	0.61	0.62
6.0–6.9	-0.0102	0.5865	0.00065	0.52	0.53	0.55	0.56	0.59	0.61	0.63	0.66	0.67
7.0–7.9	-0.0110	0.6306	0.00065	0.56	0.57	0.59	0.60	0.63	0.66	0.68	0.71	0.72
8.0–8.9	-0.0109	0.6758	0.00066	0.60	0.61	0.63	0.65	0.68	0.71	0.73	0.76	0.77
9.0–9.9	-0.0098	0.7241	0.00065	0.64	0.65	0.68	0.69	0,.72	0.76	0.78	0.81	0.83
10.0–10.9	-0.0074	0.7764	0.00065	0.69	0.70	0.73	0.74	0.78	0.81	0.83	0.87	0.88
11.0–11.9	-0.0027	0.8316	0.00064	0.74	0.75	0.78	0.80	0.83	0.87	0.89	0.92	0.94
12.0–12.9	0.0033	0.8886	0.00062	0.79	0.80	0.83	0.85	0.89	0.93	0.95	0.98	1.00
13.0–13.9	0.0090	0.9461	0.00059	0.84	0.86	0.89	0.91	0.95	0.98	1.00	1.04	1.05
14.0–14.9	0.0132	10.023	0.00055	0.90	0.91	0.94	0.96	1.00	1.04	1.06	1.09	1.11
15.0–15.9	0.0150	10.527	0.00052	0.95	0.96	1.00	1.02	1.05	1.09	1.11	1.14	1.15
16.0–16.9	0.0144	10.962	0.00048	1.00	1.01	1.04	1.06	1.10	1.13	1.15	1.18	1.19
17.0–17.9	0.0124	11.327	0.00044	1.04	1.05	1.08	1.10	1.13	1.17	1.18	1.21	1.23
18.0–18.9	0.0101	11.657	0.0004	1.08	1.09	1.12	1.13	1.17	1.20	1.21	1.24	1.25
**Females**												
4.0–4.9	0.0209	0.5280	0.00044	0.48	0.49	0.50	0.51	0.53	0.54	0.55	0.57	0.57
5.0–5.9	0.0195	0.5611	0.00044	0.51	0.52	0.53	0.54	0.56	0.58	0.59	0.60	0.61
6.0–6.9	0.0179	0.5956	0.00045	0.54	0.55	0.57	0.58	0.60	0.61	0.62	0.64	0.64
7.0–7.9	0.0164	0.6322	0.00045	0.58	0.58	0.60	0.61	0.63	0.65	0.66	0.68	0.68
8.0–8.9	0.0152	0.6710	0.00045	0.61	0.62	0.64	0.65	0.67	0.69	0.70	0.72	0.73
9.0–9.9	0.0142	0.7118	0.00045	0.65	0.66	0.68	0.69	0.71	0.73	0.75	0.76	0.77
10.0–10.9	0.0138	0.7539	0.00045	0.69	0.70	0.72	0.73	0.75	0.78	0.79	0.81	0.82
11.0–11.9	0.0139	0.7962	0.00045	0.73	0.74	0.76	0.77	0.80	0.82	0.83	0.85	0.86
12.0–12.9	0.0144	0.8361	0.00045	0.76	0.77	0.80	0.81	0.84	0.86	0.87	0.90	0.91
13.0–13.9	0.0146	0.8710	0.00044	0.80	0.81	0.83	0.84	0.87	0.90	0.91	0.93	0.94
14.0–14.9	0.0134	0.9012	0.00044	0.83	0.84	0.86	0.87	0.90	0.93	0.94	0.97	0.98
15.0–15.9	0.0101	0.9281	0.00044	0.85	0.86	0.89	0.90	0,.93	0.96	0.97	0.99	1.00
16.0–16.9	0.0051	0.9524	0.00043	0.88	0.89	0.91	0.92	0.95	0.98	1.00	1.02	1.03
17.0–17.9	-0.000	0.9744	0.00043	0.90	0.91	0.93	0.95	0.97	1.00	1.02	1.05	1.06
18.0–18.9	-0.006	0.9951	0.00042	0.92	0.93	0.95	0.97	1.00	1.02	1.04	1.07	1.08

**Table 5:** LMS values and percentile distribution of BMC (kg) of the total body of children and adolescents based on age and sex.

Age	L	M	S	P3	P5	P15	P25	P50	P75	P85	P95	P97
**Males**												
4.0–4.9	0.0053	0.2523	0.0024	0.15	0.16	0.19	0.21	0.25	0.30	0.32	0.36	0.38
5.0–5.9	0.0059	0.3914	0.0022	0.24	0.26	0.30	0.33	0.39	0.45	0.49	0.55	0.57
6.0–6.9	0.0065	0.5339	0.0021	0.34	0.36	0.42	0.46	0.53	0.61	66	0.73	0.76
7.0–7.9	0.0073	0.6802	0.0019	0.45	0.47	0.55	0.59	0.68	0.77	0.82	0.91	0.94
8.0–8.9	0.0085	0.8311	0.0016	0.56	0.60	0.68	0.73	0.83	0.93	0.98	1.08	1.11
9.0–9.9	0.0101	0.9924	0.0010	0.69	0.73	0.83	0.89	0.99	1.10	1.16	1.25	1.29
10.0–10.9	0.0120	11.673	0.0014	0.84	0.88	0.99	1.05	1.17	1.28	1.34	1.44	1.48
11.0–11.9	0.0143	13.531	0.0013	1.00	1.05	1.16	1.23	1.35	1.47	1.53	1.63	1.67
12.0–12.9	0.0167	15.455	0.0011	1.17	1.22	1.35	1.42	1.55	1.66	1.73	1.83	1.86
13.0–13.9	0.0189	17.392	0.0010	1.36	1.41	1.54	1.61	1.74	1.86	1.92	2.02	2.06
14.0–14.9	0.0204	19.228	0.0009	1.55	1.60	1.73	1.80	1.92	2.04	2.10	2.20	2.23
15.0–15.9	0.0209	20.860	0.0008	1.73	1.78	1.90	1.97	2.09	2.20	2.26	2.35	2.39
16.0–16.9	0.0207	22.234	0.0007	1.89	1.93	2.05	2.11	2.22	2.33	2.39	2.48	2.51
17.0–17.9	0.0201	23.363	0.0006	2.02	2.07	2.17	2.23	2.34	2.44	2.49	2.58	2.61
18.0–18.9	0.0194	24.378	0.0005	2.15	2.19	2.28	2.34	2.44	2.53	2.58	2.67	2.70
**Females**												
4.0–4.9	0.0084	0.4257	0.0015	0.30	0.32	0.36	0.38	0.43	0.47	0.50	0.54	0.55
5.0–5.9	0.0092	0.5189	0.0014	0.38	0.39	0.44	0.47	0.52	0.57	0.60	0.65	0.66
6.0–6.9	0.0098	0.6199	0.0013	0.46	0.48	0.53	0.56	0.62	0.68	0.71	0.76	0.78
7.0–7.9	0.0104	0.7311	0.0012	0.56	0.58	0.63	0.67	0.73	0.79	0.83	0.88	0.91
8.0–8.9	0.0109	0.8510	0.0011	0.66	0.68	0.75	0.78	0.85	0.92	0.95	1.01	1.04
9.0–9.9	0.0115	0.9770	0.0010	0.77	0.80	0.87	0.90	0.98	1.05	1.09	1.15	1.17
10.0–10.9	0.0120	11.054	0.0010	0.89	0.92	0.99	1.03	1.11	1.18	1.22	1.29	1.31
11.0–11.9	0.0126	12.324	0.0009	1.01	1.04	1.11	1.15	1.23	1.31	1.35	1.42	1.45
12.0–12.9	0.0132	13.503	0.0008	1.12	1.15	1.23	1.27	1.35	1.43	1.47	1.54	1.57
13.0–13.9	0.0135	14.515	0.0008	1.22	1.25	1.32	1.37	1.45	1.53	1.57	1.65	1.67
14.0–14.9	0.0131	15.374	0.0007	1.30	1.33	1.41	1.45	1.54	1.62	1.66	1.73	1.76
15.0–15.9	0.0116	16.123	0.0007	1.38	1.41	1.48	1.53	1.61	1.69	1.74	1.81	1.84
16.0–16.9	0.0094	16.786	0.0007	1.45	1.48	1.55	1.60	1.68	1.76	1.81	1.88	1.91
17.0–17.9	0.0068	17.369	0.0007	1.51	1.54	1.61	1.65	1.74	1.82	1.87	1.94	1.97
18.0–18.9	0.0040	17.901	0.0006	1.57	1.60	1.67	1.71	1.79	1.87	1.92	2.00	2.03

## Discussion

The initial objective of this study was to develop regression equations to predict bone health in Chilean children and adolescents based on anthropometric variables. The results confirmed that the forearm length, femur diameter, and the APVC were variables that predicted BMD and BMC in children and adolescents of both sexes.

These results are consistent with those carried out with other pediatric populations with these anthropometric variables [18–21] and biological maturation [22–24]. They have been confirmed as strong predictors of bone health. Furthermore, the findings from this study support the correlations observed between BMD and BMC and the anthropometric variables studied here. They are principally due to the BMC being dependent on bone length and diameter and bone density, respectively [25]. Therefore, the presence of short and narrow bones could lead to reduced BMD and BMC and possibly to a number of health consequences in general. However, the maximum increase in bone mass during childhood and adolescence may be achieved by means of constant changes in lifestyle that need to be implemented at an early age [26].

As a result, the forearm length, femur diameter, and APHV were used as independent variables to develop prediction models for BMD as well as for the BMC for both sexes. Therefore, the four models proposed showed a high precision in their regression coefficients as described in the literature [27,28]. Thus, the coefficients of determination showed an explanatory power between 0.76 and 0.84%. Furthermore, the SEE were less than 0.29, and the variance inflation factor (VIF) showed ranges of less than values established as normal (>0.10, <10.0) as described by Slinker and Glantz [29].

On the other hand, no significant differences occurred between the mean reference values (DXA) and the mean values estimated by the four predictor models. In addition, the four proposed equations showed a good agreement (Plotting of Bland-Altman) with the DXA referencing method since the limits of 95% are narrow and the correlation coefficients are highly significant.

Furthermore, the desired reproducibility indicator (IRD) was used with the goal of clearly defining of the measurements. This indicator evaluates the agreement between two readings of the same sample based on the concordance correlation coefficient (CCC) in terms of precision and accuracy [15]. Therefore, the complete collection of values of the indicators shows from good to excellent agreement: CCC = 0.86 to 0.99 [15, 30] for all four equations.

In essence, these results support the reproducibility of the proposed equations because they represent another step in n observing equivalent instances between methods since the use of CCC ensures precision and accuracy of the theoretical results [31]. This illustrates and guarantees the robustness of the models developed in this study.

Therefore, based on the four equations for estimating bone health, percentiles were developed for each age and sex. This contribution is a non-invasive alternative that serves to assess and detect early on reduced levels of BMD and BMC. This is essential and necessary for developing intervention programs [32], especially for children and adolescents with low levels of bone mass [33].

In general, curves have been developed based on a number of international studies to assess bone health in pediatric samples from various countries around the world [22,30,34–36]. For the evaluations, these international studies used sophisticated and costly equipment. However, to our knowledge, it is clear to us that to date no studies have published references based on anthropometric variables. Therefore, these reference curves dramatically reduce costs. Moreover, their use and implementation may be advantageous for health clinics and educational institutions where resources and infrastructures are limited.

In this study, use of percentiles based on simple anthropometric variables and controlling for somatic maturation through APHV could serve to help professionals and researchers improve the bone health care of children and adolescents. Furthermore, this could help compare and classify children according to established cut-off points (normal, osteopenia, and osteoporosis). These reference values should show practical applications for detecting skeletal anomalies in children and adolescents.

It is necessary to point out that in this study, somatic maturation was controlled. Chronological age may not be the best indicator of growth and development. Therefore, APHV has been introduced into the calculations to control for the somatic maturation rhythms, and in this way, appropriate estimations may be made for adolescents experiencing delayed, normal, or accelerated maturation.

To summarize, the present data generated meet the conditions established by the International Society of Clinical Densitrometry (ISCD). It recommends using a sample sufficiently large of the general population in order to include gender, age, and ethnic origin [37]. Moreover, this study has taken into account the fidelity of the measurements and the adequate use of a statistical model to generate the percentiles (LMS method) as suggested by Butte et al. [38].Therefore, the focus of the LMS modeling offers a greater precision in describing reference ranges, in particular those superior and inferior extremes in distribution [39].

This study demonstrated some strengths. One of these was the exclusion of the head from the total body scan. In general, the head is disproportionally large in small children, and it can mask deficiencies in other places in the skeleton. Furthermore, the large sample size included non-smokers not suffering from previous fractures, and not taking additional vitamin supplements. Moreover it is a representative sample of healthy children and adolescents. Therefore, the results presented here may be used daily to determine bone health similar to those found on the following link: www.http://reidebihu.net/saludosea.php

For future studies, it is necessary to take into account the clinical history and medication use of the subjects, especially important in some specific cases. However, in this study, the information was not available for the entire sample. Therefore, it was not taken into consideration for the 3020 subjects studied.

## Conclusion

Four new accurate and precise equations were developed to estimate BMD and BMC of Chilean children and adolescent. Furthermore, reference norms were proposed to monitor bone health by age and sex based on APHV.

These non-invasive instruments help identify children with potential underlying problems in bone mineralization during the growth stage and biological maturation. We suggest using and implementing the results in clinical and epidemiological contexts during childhood and adolescence.
